# Heme Oxygenase 1-Mediated Anti-Inflammatory Effect of Extract from the Aerial Part of *Heracleum moellendorffii* Hance

**DOI:** 10.3390/foods12173309

**Published:** 2023-09-02

**Authors:** Hyun Young Jang, Syng-Ook Lee

**Affiliations:** Department of Food Science and Technology, Keimyung University, Daegu 42601, Republic of Korea

**Keywords:** *Heracleum moellendorffii* Hance, HO-1, inflammation, leaves, Nrf2

## Abstract

In this study, the anti-inflammatory effects of a methanolic extract from the aerial part of *Heracleum moellendorffii* Hance (HmAPE) and its underlying mechanisms were investigated. HmAPE demonstrated a significant reduction in nitric oxide production in lipopolysaccharide (LPS)-treated murine macrophage RAW264.7 cells, and HmAPE decreased the protein and mRNA expression of inducible nitric oxide synthase. Further mechanistic studies on inflammatory signaling pathways revealed that HmAPE-mediated downregulation of inflammatory gene expressions was not associated with mitogen-activated protein kinases or nuclear factor-κB signaling pathways. However, HmAPE treatment activated nuclear factor E2-related factor 2 (Nrf2) and upregulated heme oxygenase-1 (HO-1) expression, which is known to suppress pro-inflammatory cytokine production. Additionally, treatment with a selective HO-1 inhibitor, tin protoporphyrin IX, partially reversed the effects of HmAPE in LPS-treated RAW264.7 cells, indicating that HmAPE inhibited LPS-induced NO production, at least in part, through induction of Nrf2-mediated HO-1 expression. These findings suggest that HmAPE could serve as a potential edible source with anti-inflammatory properties, and further studies are required to ascertain its anti-inflammatory efficacy in vivo.

## 1. Introduction

Inflammation stands as a critical immune response, serving to protect the body from infections and tissue damage. In the presence of inflammatory triggers, macrophages become activated, releasing significant amounts of pro-inflammatory cytokines such as nitric oxide (NO), tumor necrosis factor-α (TNF-α), interleukin-1β (IL-1β), and prostaglandin E2 [1]. Proper control of inflammation is essential to prevent tissue damage, as uncontrolled inflammation can lead to various inflammation-mediated chronic diseases, such as arthritis, inflammatory bowel disease, cancer, cardiovascular disease, and diabetes [2]. Therefore, regulating pro-inflammatory mediators is of utmost importance in preventing and treating inflammatory diseases.

The inflammatory process involves two major transcription factors: nuclear factor-κB (NF-κB) and nuclear factor erythroid 2-related factor 2 (Nrf2). NF-κB serves as a pivotal transcription factor, governing the expression of pro-inflammatory genes such as inducible nitric oxide synthase (iNOS), cyclooxygenase-2 (COX-2), and TNF-α, and NF-κB and its upstream signaling moleucles, including extracellular signal-regulated kinase 1/2 (ERK1/2), c-jun N-termainal kinases (JNKs), and p38 mitogen-activated protein kinases (p38) get activated by inflammatory stimuli, such as bacterial lipopolysaccharide (LPS). On the other hand, Nrf2 is a pivotal transcription factor modulating the expression of anti-inflammatory genes, such as heme oxygenase-1 (HO-1). HO-1 is an inducible enzyme that facilitates the breakdown of heme into biliverdin, carbon monoxide, and ferrous iron. Furthermore, HO-1 inhibits iNOS expression, thereby suppressing NO production. Numerous studies have demonstrated that the Nrf2-mediated upregulation of HO-1 prevents the production of various pro-inflammatory mediators in LPS-treated macrophages [3,4,5].

For hundreds of years, plant extracts and plant-derived compounds have been utilized as folk remedies to address inflammation [6], and their safety and effectiveness have been proven for a long time. Consequently, phytochemicals have garnered considerable attention for their anti-inflammatory effects [6]. The Heracleum genus, belonging to the dropwort family and comprising nearly 125 species worldwide, is one of the largest genera of Umbelliferae (Apiaceae). Among these species, *Heracleum moellendorffii* Hance grows in fields and mountains across several countries in Asia [7]. The roots of *H. moellendorffii* Hance have a history of traditional use as an herbal remedy for treating inflammatory diseases such as arthritis, backache, and fever [8]. Numerous compounds including coumarins, monoterpenoids, polyacetylenes, and sesquiterpenoids have been isolated from the roots [9,10]. Furthermore, the essential oil derived from the roots and aerial parts of *H. moellendorffii* Hance has demonstrated insecticidal activity [11]. In Korea, the young leaves of *H. moellendorffii* Hance have been consumed as an edible wild herb, and previous studies have reported a range of pharmacological activities of the leaves, including antioxidant and anti-melanogenic effects [8,12]. However, to date, no study has been conducted to investigate the anti-inflammatory activities and mechanisms of the young leaves of *H. moellendorffii* Hance.

Therefore, the present study aimed to investigate the anti-inflammatory impacts and the underlying mechanisms attributed to the phenolic extract of the aerial part of *H. moellendorffii* Hance (HmAPE) in LPS-treated RAW264.7 macrophages.

## 2. Materials and Methods

### 2.1. Preparation of H. moellendorffii Hance Extracts

The young aerial parts of *H. moellendorffii* Hance were obtained from Chamjoheun Igija Farm (Hwacheon-gun, Gangwondo, Hwacheon-gun, Republic of Korea). To prepare polyphenol-rich extract [13,14], a total of 100 g of the aerial parts and roots of *H. moellendorffii* Hance were extracted three times using 80% methyl alcohol (*v*/*v*) containing 0.2% HCl (900 mL) at 45 °C for 24 h. Subsequently, the extracts underwent filtration, concentration under reduced pressure, and lyophilization. The yields of the phenolic extract (HmAPE) and root extract (HmRE) were found to be 12.17% and 13.29%, respectively.

### 2.2. Cell Culture

Dulbecco’s Modified Eagle Medium (DMEM) complemented with 10% fetal bovine serum, 100 units/mL of penicillin, and 100 mg/mL of streptomycin was used to cultivate RAW264.7 murine macrophages (KCLB, Seoul, Republic of Korea). The cells were maintained at 37 °C within a humidified CO_2_ incubator (5% CO_2_), and once the cells reached 70% to 80% confluence, they were harvested using a cell lifter and subcultured.

### 2.3. Cell Viability Assay

To determine cell viability, a 3-(4,5-dimethylthiazol-2-yl)-2,5-diphenyltetrazolium bromide (MTT) assay was used. The MTT assay relies on converting a yellow tetrazolium salt (MTT) into purple formazan crystals by viable cells. Living cells harbor NAD(P)H-dependent oxidoreductase enzymes that facilitate the transformation of MTT into formazan [15]. Cells were initially seeded at a density of 3 × 10^5^ cells/well in 48-well plates and allowed to incubate overnight. Following 24 h at 37 °C, the cells were pre-incubated with a fresh medium containing HmAPE for 1 h before exposure to LPS (100 ng/mL; Sigma-Aldrich, catalog number L4391, St. Louis, MO, USA) for 20 h at 37 °C. After the treatment, 20 μL of MTT solution (2.5 mg/mL) was introduced into each well, followed by an additional 4-h incubation. The formazan formed was dissolved in 250 μL of DMSO, and the absorbance at 570 nm was measured using a microplate reader (Epoch; Biotek Instruments, Winooski, VT, USA).

### 2.4. Quantification of NO

NO production was assessed by measuring the levels of nitrite, the stable degradation products of NO, in the medium using the Griess Reagent System, as previously described [16]. Briefly, the cells were pre-incubated with HmAPE for 1 h and then treated with LPS (100 ng/mL) for 24 h. Culture media (50 μL/well) were then mixed with 50 μL of Griess reagent, followed by a 10-min incubation within a darkened chamber. Subsequently, the absorbance at 540 nm was measured using a microplate reader, and the outcomes were compared against a standard calibration curve prepared from sodium nitrite (NaNO_2_).

### 2.5. Quantitative Real-Time Polymerase Chain Reaction (qPCR) and Western Blot Analyses

qPCR and western blot analyses were conducted following the procedures described in a previous study [17]. To conduct western blotting, whole-cell lysates were prepared using RIPA buffer (Thermo, Waltham, MA, USA) supplemented with phosphatase and protease inhibitors. The protein concentration of the lysates was determined using a BCA protein assay kit (Thermo), and for electrophoresis, 25 μg of cellular proteins were separated on a 10% SDS-polyacrylamide gel (PAGE). Polyclonal antibodies targeting HO-1 (#374090) and Nrf2 (sc-722) were procured from Merck (Kenilworth, NJ, USA) and Santa Cruz Biotechnology (Santa Cruz, CA, USA), respectively, and monoclonal antibodies against iNOS (#13120), p65 (#8242), phospho-p65 (p-p65; #3033), p-ERK1/2 (#4370), p-JNK (#4668), p-p38 (#4511), and glyceraldehyde 3-phosphate dehydrogenase (GAPDH; #2118) were procured from Cell Signaling Technology (Danvers, MA, USA). All primary antibodies were used at a dilution of 1:1000. Total RNA for qPCR analysis was isolated using TRIzol reagent (Ambion Inc., Carlsbad, CA, USA) by the manufacturer’s guidelines. cDNA was synthesized from the extracted total RNA using the Reverse Transcription System (Takara Bio Inc., Shiga, Japan). Each PCR was performed in triplicate, within a 20-μL volume, using SYBR Green Premix (Takara Bio Inc., Shiga, Japan). The thermal cycling conditions comprised an initial denaturation at 95 °C for 15 min, followed by 40 cycles of 95 °C for 30 s and 60 °C for 1 min, all conducted using a real-time PCR machine (Takara). In each PCR amplification, a thermal protocol consisting of 95 °C for 15 s, 60 °C for 15 s, and a gradual increase to 95 °C was applied. The primer sequences used for qPCR are listed in Table 1.

### 2.6. Subcellular Localization Assay

The subcellular localization assay was performed as previously described [18]. Cells (2 × 10^5^ cells/well) were seeded onto 12 mm coverslips in 12-well plates and subsequently treated with either the vehicle control (DMSO), HmARE, or sulforaphane (SFN). After 6 h, the cells were fixed using 4% paraformaldehyde (Sigma Aldrich, St. Louis, MO, USA) in PBS (pH 7.4) for 15 min. Following PBS washing, permeabilization was achieved by immersing the cells in a 0.3% Triton X-100 solution in PBS for 10 min, followed by blocking with normal goat serum (Vector Lab., Burlingame, CA, USA). Subsequently, the cells were incubated with anti-Nrf2 rabbit IgG (Cell Signaling, Beverly, MA, USA), followed by incubation with anti-rabbit IgG conjugated with FITC (Life Technology, Eugene, OR, USA). The cells were mounted using a mounting medium containing DAPI (Abcam, Cambridge, MA, USA) for nuclear counterstaining. Fluorescent images were captured using a Zeiss Axioskop 50 fluorescence microscope (Carl Zeiss, Jena, Germany) equipped with a ProgRes C5 camera (Jenoptik, Jena, Germany).

### 2.7. High Performance Liquid Chromatography (HPLC) Analysis

HPLC analysis was conducted employing a Shimadzu 06959 series HPLC system (Shimadzu Corp., Kyoto, Japan) with a UV detector. Data analysis was accomplished using LC solution software (version 1.24). A SunFire C^18^ column (250 mm, pore size 5 μm; Waters, Milford, MA, USA) was employed as an analytical column. The conditions for HPLC analysis are shown in Table 2. HmAPE and a standard compound (hyperoside) were dissolved in methanol (5 mg/mL) and filtered with a 0.45 μm membrane filter before subjecting them to HPLC analysis.

### 2.8. Statistical Analysis

Statistical significance was assessed using a *t*-test (Sigma Plot 10.0, Systat Software, Inc., San Jose, CA, USA) or a one-way ANOVA followed by Duncan’s multiple range test (SPSS 23.0, SPSS Inc., Chicago, IL, USA). The outcomes are displayed as the mean along with the standard error of the mean (SEM) across three experiments, and a *p*-value below 0.05 was deemed to indicate statistical significance.

## 3. Results

### 3.1. The HPLC Analysis of HmAPE

**Figure 1:**
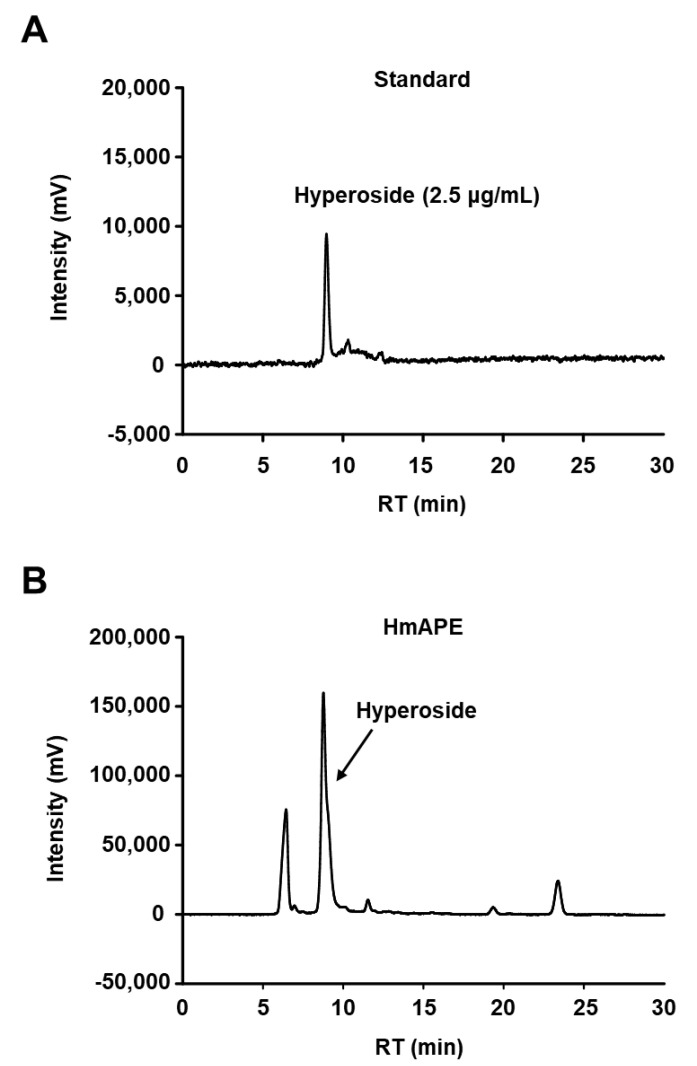
HPLC chromatograms of hyperoside and HmAPE. Chromatograms of hyperoside standard (**A**) and HmAPE (**B**).

Hyperoside (quercetin-3-O-β-D-galactoside pyranose) is a flavonoid that is abundantly present in Heracleum species, including *H. moellendorffii* Hance [8,19]. The phytochemical composition of HmAPE was analyzed using HPLC with hyperoside as a standard component. As previously reported, hyperoside was identified as a major compound in the HmAPE used in this study (Figure 1).

### 3.2. Anti-Inflammatory Effects of HmAPE in LPS-Treated RAW264.7 Cells

**Figure 2:**
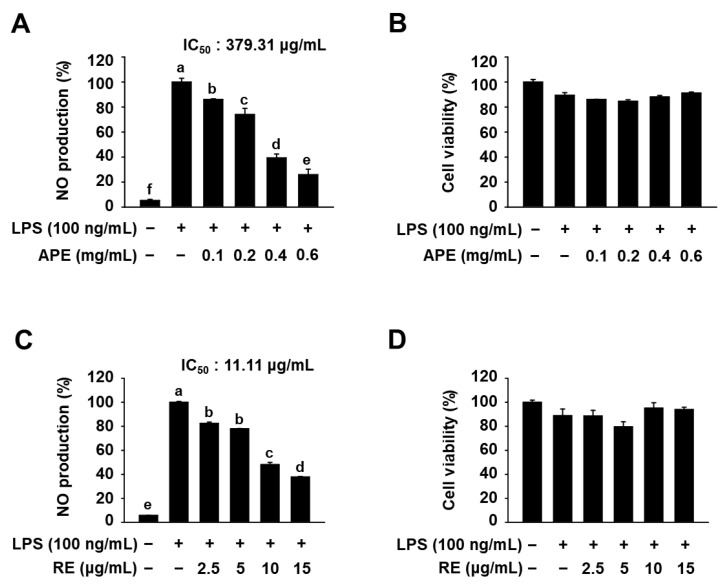
Effects of HmAPE and HmRE on NO production and cell viability in LPS-treated RAW264.7 cells. Cells were pre-incubated with indicated concentrations of HmAPE or HmRE for 1 h and then treated with LPS for 24 h. NO production (**A**,**C**) and cell viability (**B**,**D**). The results were represented as the mean ± SEM (*n* ≥ 3). A different letter indicates a significant difference among groups (*p* < 0.05).

To investigate the anti-inflammatory effects of HmAPE, we first examined whether HmAPE could modulate NO production in LPS-treated RAW264.7 cells. As depicted in Figure 2A, LPS treatment caused a significant increase in NO production, whereas HmAPE dose-dependently inhibited NO production (IC50 = 379.31 μg/mL). Additionally, HmAPE, at any of the concentrations tested along with LPS, did not exhibit significant cytotoxic effects compared to cells treated with LPS alone (Figure 2B). This suggests that the inhibitory effect of HmAPE on NO production was not due to cytotoxicity in RAW264.7 cells. Notably, a previous study [19] demonstrated that the root extract of *H. moellendorffii* Hance (HmRE) also suppressed NO production in LPS-treated cells. This study confirmed a significant inhibition of LPS-induced NO production by the root extract of *H. moellendorffii* Hance (HmRE), with an IC50 value of 11.11 μg/mL, which is much lower than that of HmAPE (Figure 2C,D).

**Figure 3:**
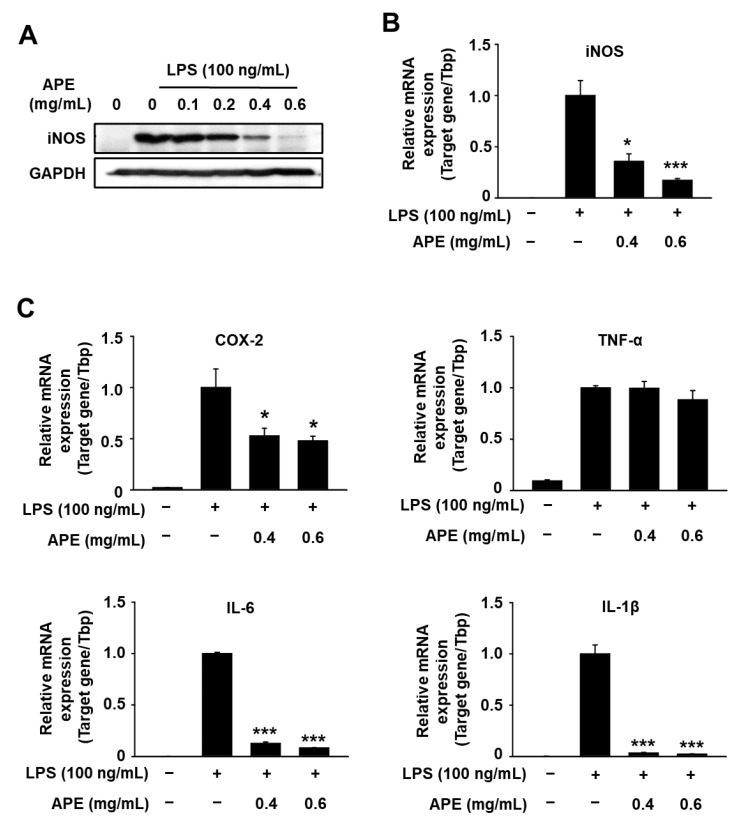
Effects of HmAPE on the expression of iNOS and pro-inflammatory cytokines in LPS-treated RAW264.7 cells. Cells (1.5 × 10^5^ cells/well) were pre-incubated with APE for 1 h and then treated with LPS for 24 h (protein expression) or 18 h (mRNA expression). Whole-cell lysates were subjected to western blot analysis, with GAPDH employed as a loading control (**A**). mRNA expression levels were quantified through qPCR, utilizing TbP as an internal reference (**B**,**C**). The results were represented as the mean ± SEM (*n* ≥ 3). * *p* < 0.05, and *** *p* < 0.005 vs. the LPS control.

Considering that LPS-stimuated NO production in macrophages is acknowledged to be regulated by iNOS, we examined the influence of HmAPE on the modulation of iNOS gene expression. As anticipated, treatment of RAW264.7 cells with LPS significantly increased mRNA and protein levels of iNOS. However, this increase was significantly inhibited by HmAPE (Figure 3A,B). These findings indicate that HmAPEinhibits NO production in LPS-treated cells by downregulating iNOS gene expression.

Moreover, we analyzed the effects of HmAPE on the expression of various other inflammatory cytokines using qPCR analysis in LPS-treated cells. The results demonstrated that HmAPE suppressed mRNA expression of IL-1β, IL-6, and COX-2, whereas the TNF-α mRNA level did not show significant changes (Figure 3C). These results suggest that HmAPE has the potential to regulate inflammation by inhibiting NO production and suppressing the gene expression of various inflammatory mediators.

### 3.3. Anti-Inflammatory Mechanisms of HmAPE: Effect on the MAPKs/NF-κB Signaling Pathways

**Figure 4:**
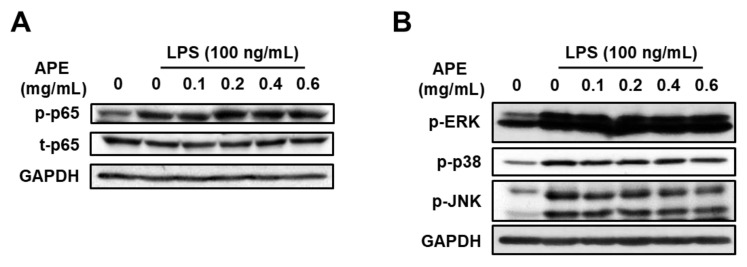
Effects of HmAPE on NF-κB and MAPKs signalings in LPS-treated RAW264.7 cells. Cells (3 × 10^5^ cells/well) were pre-incubated with HmAPE for 1 h and then treated with LPS for 30 min (**A**) or 20 min (**B**).

A study by Kim et al. [20] demonstrated that HmRE exhibited an anti-inflammatory effect by inhibiting the MAPKs/NF-κB signaling pathways. To investigate whether HmAPE affected MAPKs/NF-κB signaling, we analyzed the phosphorylation (activation) of p65 and MAPKs using western blotting. LPS treatment increased the levels of p-p65, p-JNK, and p-p38. However, HmAPE treatment did not lead to significant changes in the levels of p-p65 or p-MAPKs in LPS-treated RAW264.7 cells (Figure 4). This indicates that HmAPE-mediated inhibition of NO production and the expression of pro-inflammatory cytokines is not associated with the regulation of MAPKs/NF-κB signaling pathways. Therefore, HmAPE appears to regulate inflammatory responses through different mechanisms than HmRE. Consequently, we further investigated the effect of HmAPE on HO-1, a well-known inflammatory modulator.

### 3.4. Anti-Inflammatory Mechanisms of HmAPE: Effect on the Nrf2/HO-1 Signaling Pathway

**Figure 5:**
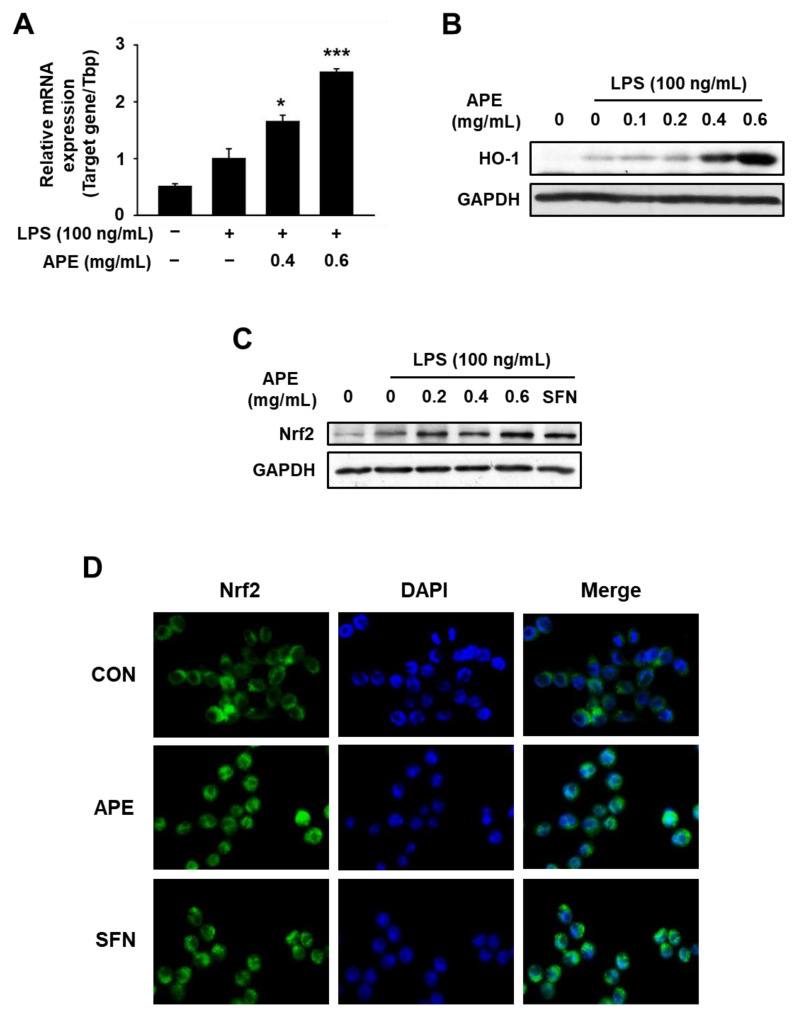
Effects of HmAPE on the expression of HO-1 and Nrf2 in LPS-treated RAW264.7 cells. Cells (3 × 10^5^ cells/well) were pre-incubated with HmAPE for 1 h and then treated with LPS for 18 h (**A**), 24 h (**B**), or 6 h (**C**,**D**). mRNA expression levels were determined by qPCR, and TbP was used as an internal control (**A**). Sulforaphane (SFN; 10 μM) was used as a positive control (**D**). The results were represented as the mean ± SEM (*n* ≥ 3). * *p* < 0.05 and *** *p* < 0.005 vs. the LPS control.

To clarify whether HmAPE affected HO-1 expression, we analyzed its mRNA and protein levels in cells treated with LPS in the presence of HmAPE. The results demonstrated that LPS treatment slightly increased the mRNA and protein levels of HO-1, while HmAPE treatment significantly and dose-dependently elevated these levels (Figure 5A,B).

Next, to elucidate the mechanism behind HmAPE-induced HO-1 induction, RAW264.7 cells were exposed to LPS for 6 h in the presence of HmAPE. The subsequent analysis involved the evaluation of Nrf2 protein expression through western blotting and assessing its cellular localization via immunofluorescence staining. As shown in Figure 5C, Nrf2 expression was significantly enhanced upon HmAPE treatment. Immunostaining showed that HmAPE or sulforaphane (SFN; a known Nrf2 activator) led to the translocation of Nrf2 into the nucleus (Figure 5D).

**Figure 6:**
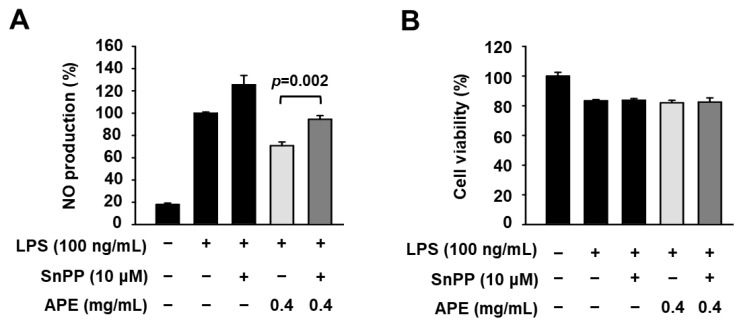
Effects of SnPP on NO production and cell viability in LPS-treated RAW264.7 cells. Cells were pre-incubated with HmAPE for 1 h in the presence of SnPP (10 μM) and then treated with LPS for 24 h. NO production (**A**) and cell viability (**B**). The results were represented as the mean ± SEM (*n* ≥ 3).

To further confirm whether the HmAPE-mediated decrease in NO production is related to the induction of HO-1 by HmAPE, the association between these factors was investigated using the HO-1 inhibitor tin protoporphyrin IX (SnPP). Interestingly, SnPP treatment partly but significantly reversed the HmAPE-mediated decrease in NO production (Figure 6).

## 4. Discussion

Macrophages play a crucial role in regulating inflammation and various immune responses in the host immune system, and they are commonly activated by LPS [21]. NO, a major pro-inflammatory mediator produced by macrophages, serves as a signaling molecule that regulates several physiological functions, such as neurotransmission, vasodilation, and anti-microbial and cytoprotective effects. However, high concentrations of NO can lead to inflammatory diseases and DNA damage due to its free radical and weak oxidant properties [22]. Therefore, the regulation of NO production is considered a valuable strategy for treating inflammatory disorders.

NO production is catalyzed by NOS through the oxidation of L-arginine, and NOS consists of three isoenzymes: endothelial NOS (eNOS), iNOS, and neuronal NOS (nNOS). eNOS is constitutively expressed in vascular endothelial cells and nNOS is expressed in central and peripheral neurons. These two enzymes are low-output enzymes that play essential roles in the vascular and nervous systems, respectively. On the other hand, iNOS, which is not typically expressed in cells, can be induced by various inflammatory stimuli. Once expressed, iNOS remains constantly active and unregulated, leading to the production and release of large amounts of NO that can aggravate inflammatory disorders such as inflammatory bowel diseases, rheumatoid arthritis, and septic shock [23].

Notably, a previous study [20] demonstrated that the root extract of HmRE also suppressed NO production through inhibition of iNOS expression in LPS-treated RAW264.7 cells. The results derived from this study (Figure 2A and Figure 3A,B) also indicate that HmAPE inhibits NO production in LPS-treated cells by downregulating iNOS gene expression, and these results and Figure 3C imply that HmAPE holds promise in regulating inflammation by inhibiting NO production and repressing the gene expression of diverse inflammatory mediators.

MAPKs and NF-κB are crucial intracellular signaling molecules that mediate the synthesis and secretion of various inflammatory cytokines in LPS-treated macrophages [24]. NF-κB plays a pivotal role in regulating the expression of genes involved in inflammation. In the absence of stimuli, NF-κB, comprising its p50 and p65 subunits, remains bound to the inhibitory protein, IκB, in the cytoplasm. Upon cellular stimulation, IκB undergoes rapid phosphorylation, leading to its degradation. The released NF-κB then translocates from the cytosol to the nucleus, inducing downstream genes by binding to the NF-κB response element [25]. MAPKs (ERK1/2, JNK, and p38) constitute a family of critical signaling proteins that contribute to the production of pro-inflammatory cytokines [1]. The phosphorylation of MAPKs is closely involved in regulating NF-κB activation [26,27]. Consequently, inhibiting the activation of NF-κB and MAPKs is considered a key target for anti-inflammatory agents.

HO-1 has also gained attention as an antioxidant and anti-inflammatory enzyme. Heme is a critical component of iNOS, and the activation of HO-1 can limit heme availability for optimal iNOS activity or synthesis [28]. Additionally, CO, a breakdown product of heme, inhibits the transcription and activity of iNOS by binding to the heme moiety of the enzyme [6]. Moreover, HO-1 and its catalytic products possess significant anti-inflammatory effects. Nrf2, a pivotal transcription factor that governs HO-1 gene expression, resides typically within the cytoplasm as part of a heteromeric complex with Kelch-like ECH-associated protein (Keap) 1, a cytosolic repressor of Nrf2. Upon stimulation by various extracellular stimuli, Nrf2 dissociates from Keap1. Consequently, liberated Nrf2 undergoes activation and translocation into the nucleus. Within the nucleus, activated Nrf2 binds to the antioxidant response element, prompting the upregulation of the anti-inflammatory and antioxidant enzyme HO-1 [29]. Indeed, numerous extracts and phytochemicals derived from natural products such as red ginseng [30], mushroom *Ganoderma lucidum* [31], *Selaginella tamariscina* [32], and *Taraxacum officinale* [33] have been reported to regulate inflammatory responses through the activation of NRF2/HO-1 pathway in LPS or lipoteichoic acid-stimulated macrophages.

A previous study [20] demonstrated that HmRE exhibited an anti-inflammatory effect by inhibiting the MAPKs/NF-κB signaling pathways and inducing HO-1 expression. However, in LPS-treated RAW264.7 cells, treatment with HmAPE did not affect MAPKs/NF-κB signaling pathways (Figure 4A,B) but activated Nrf2 (Figure 5C,D) and induced HO-1 expression (Figure 5A,B). Some studies link the regulation of MAPK signaling pathways to the activation of Nrf2 [26]. Still, there was no effect on MAPK signaling by treatment with HmAPE, indicating that HmAPE activates Nrf2 independently to MAPKs. Moreover, under the presence of the HO-1 inhibitor, SnPP, it was observed that the impact of HmAPE on NO production was partially attenuated (Figure 6). These results indicate that the increase in NO production by HmAPE treatment was at least partly dependent on increasing HO-1 activity without affecting MAPKs/NF-κB signaling pathways in LPS-treated RAW264.7 macrophages. HO-1 has also been reported to restrict the excess production of pro-inflammatory mediators such as COX-2, IL-1β, and IL-6 [34]. Consistently, the expression patterns of HO-1 and pro-inflammatory cytokines were precisely opposite (Figure 5). Thus, the decrease in the expression of COX-2, IL-1β, and IL-6 in HmAPE-treated cells may also be related to HO-1 activation.

Many plant extracts have been found to possess anti-inflammatory effects via both MAPKs/NF-κB and Nrf2/HO-1 pathways. Among them, extracts from the aerial part of various edible plants, such as *Peucedanum japonicum* Thunberg [35], *Piper betle* Linn. [36], and dandelion (*Taraxacum officinale*) [33], have shown the ability to suppress inflammatory responses via both pathways. Similarly, HmRE has been reported to inhibit the synthesis of pro-inflammatory mediators by inhibiting MAPKs/NF-κB signaling and activating Nrf2/HO-1 signaling in LPS-treated RAW264.7 cells [20]. Moreover, hyperoside, a flavonoid found in the aerial parts of *H. moellendorffii* Hance, has demonstrated anti-inflammatory activity by suppressing NF-κB activation in LPS-treated mouse peritoneal macrophages [37]. In contrast, HmAPE, derived from the aerial part of *H. moellendorffii* Hance, did not influence MAPKs/NF-κB signaling pathways but exhibited anti-inflammatory effects through the induction of the Nrf2/HO-1 pathway. These results suggest that HmAPE might contain compounds that have opposite effects on the MAPKs/NF-κB pathways. Therefore, further investigation is needed to identify and characterize the anti-inflammatory compounds present in HmAPE for future research.

With increasing interest in health, the role of functional foods has become particularly significant, and the market for these foods continues to expand each year. Consequently, research exploring the physiological activities of diverse natural substances, with a focus on addressing health-related issues is actively underway. However, the development of functional foods utilizing these materials faces limitations due to various issues such as safety concerns, lack of scientific evidence, unclear mechanisms of action, and sourcing of ingredients. Safety concerns, especially with non-edible herbal materials, are frequently highlighted, leading to a recent focus on evaluating physiological activities in edible materials to address safety issues. The young leaves of *H. moellendorffii* Hance, traditionally consumed as spring vegetables in Korea, are recognized as a food ingredient. Therefore, these leaves hold promise for the development of functional foods due to their relative freedom from safety concerns. If the anti-inflammatory activity is verified through further research involving animal models and human subjects, these young leaves could be anticipated to hold significant value as a new material capable of regulating inflammation, known as a critical factor in various chronic diseases.

## 5. Conclusions

The present study demonstrated that treatment with HmAPE inhibits inflammatory responses, in part, through Nrf2-mediated induction of HO-1 in LPS-treated RAW264.7 cells without affecting MAPKs/NF-κB signaling pathways. This study provides the first evidence for the anti-inflammatory effects of the aerial part of *H. moellendorffii* Hance, and the findings suggest that HmAPE holds promise as a nutraceutical or potential treatment option for chronic inflammation. Further investigations are needed to validate the anti-inflammatory effects in animal models of inflammation associated with respiratory diseases, gastrointestinal diseases, arthritis, etc., which could open up new avenues for potential therapeutic applications.

## Figures and Tables

**Table 1:** The primer sequences used for qPCR.

Target Gene		Primer Sequences
Tbp	Sense	5′-GTGAAGGGTACAAGGGGGTG-3′
	Antisense	5′-ACATCTCAGCAACCCACACA-3′
Nos2	Sense	5′-GTCGATGTCATGCAGCTT-3′
	Antisense	5′-GAAGAAAACCCCTTGTGCTG-3′
Cox-2	Sense	5′-GGCGCAGTTTATGTTGTCTG-3′
	Antisense	5′-CAGCACTTCACCCATCAGTT-3′
Il-1β	Sense	5′-AGGTCAAAGGTTTGGAAGCA-3′
	Antisense	5′-TGAAGCAGCTATGGCAACTG-3′
Il-6	Sense	5′-TCTGAAGGACTCTGGCTTTG-3′
	Antisense	5′-GATGGATGCTACCAAACTGGA-3′
Tnf-α	Sense	5′-GGTCTGGGCCATAGAACTGA-3′
	Antisense	5′-CAGCCTCTTCTCATTCCTGC-3′
Ho-1	Sense	5′-ACAACCAGTGAGTGGAGCCT-3′
	Antisense	5′-TCAAGGCCTCAGACAAATCC-3′

Tbp; TATA-binding protein, Nos2; iNOS.

**Table 2:** Chromatographic conditions for HPLC.

Instrument	Condition
Column	SunFire C18 (250 × 4.6 × 5 μm)
Injection volume	10 μL
Detector wavelength	280 nm
Flow rate	0.8 mL/min
Column temperature	30 °C
Mobile phase	MeOH containing 0.1% TFA

TFA; trifluoroacetic acid.

## Data Availability

The data used to support the findings of this study can be made available by the corresponding author upon request.
